# Hemoglobin A1c Levels Associated with Age and Gender in Taiwanese Adults without Prior Diagnosis with Diabetes

**DOI:** 10.3390/ijerph18073390

**Published:** 2021-03-25

**Authors:** Shih-Hao Huang, Peng-Ju Huang, Jhong-You Li, Yu-De Su, Cheng-Chang Lu, Chia-Lung Shih

**Affiliations:** 1 Department of Orthopedics, Kaohsiung Medical University Hospital, Kaohsiung Medical University, Kaohsiung 807, Taiwan; aroc12@yahoo.com.tw (S.-H.H.); roger01@ms4.hinet.net (P.-J.H.); 2 Department of Orthopedics, Kaohsiung Municipal Siaogang Hospital, Kaohsiung Medical University, Kaohsiung 812, Taiwan; mark163333@gmail.com (J.-Y.L.); u9401112@gmail.com (Y.-D.S.); 3 Research Center for Environmental Medicine, Kaohsiung Medical University, Kaohsiung 807, Taiwan; 4 College of Medicine, Kaohsiung Medical University, Kaohsiung 807, Taiwan; 5 Clinical Medicine Research Center, Ditmanson Medical Foundation Chia-Yi Christian Hospital, Chia-Yi City 600, Taiwan

**Keywords:** age, correlation, HbA1c, sex

## Abstract

Several studies have reported that Hemoglobin A1c (HbA1c) levels increase with age for people without diabetes. However, HbA1c levels associated with age and gender have not been well investigated for Taiwanese adults. The objective of this study was to investigate the sex-specific association between HbA1c levels and age for Taiwanese adults without diabetes. The data were collected from the Taiwan Biobank database with inclusive criteria being participants without diabetes. The association between HbA1c values and age was conducted by linear regression analysis, HbA1c values between sexes were compared by two-sample *t*-test, and HbA1c levels among age groups were compared using one-way ANOVA. The results showed that HbA1c levels were positively correlated with age, and the levels for males were significantly higher than for females among all participants. However, there was no significantly positive correlation between HbA1c levels and age in males for age group of 50–70 years. The levels of males were significantly higher than females for age groups of 30–39 and 40–49 years. There were significant differences in HbA1c levels among age groups for all participants, males, and females except for the two age groups of 50–59 and 60–70 years in males. Age and gender were important factors affecting HbA1c levels. Our results suggested that the HbA1c cut-point levels for the diagnosis of diabetes should vary by age and gender.

## 1. Introduction

Hemoglobin A1c (HbA1c) is an indication of chronic glycaemia and it can reflect an integrated index of glycaemia over the past 120-day lifespan of the red blood cell. The HbA1c test can be used to diagnose diabetes in which a level of 6.5% is suggested as the cut point for the diagnosis of diabetes [1]. HbA1c has been suggested to be a better indicator for controlling blood glucose levels in patients with diabetes than fasting blood sugar levels [2]. The World Health Organization reported that a total of 422 million adults globally were suffering from diabetes in 2014 [3]. It was reported that 1.5 million people died of diabetes worldwide and 2.2 million patients died of cardiovascular and other diseases that were related to higher blood glucose levels, in 2012 [3].

Several studies have reported that HbA1c levels increase with age for people without diabetes [4,5,6,7]. This relationship seems to reflect the changes in glucose tolerance related with age [8]. However, the current goals in the treatment of diabetes often do not consider the impact of age on the given HbA1c target levels [9,10]. The reference range of HbA1c adopted in most laboratories is driven from a relatively small number of people younger than 40 years of age [11]. Age and gender were suggested to be important factors affecting HbA1c levels for Chinese adults [12], and HbA1c levels between Chinese and Taiwanese adults might be different due to the differences in lifestyle. To the best of our knowledge, HbA1c levels associated with age and gender have not been well investigated for Taiwanese adults.

The objective of this study was to investigate the sex-specific association between HbA1c levels and age for Taiwanese adults without diabetes. These results could provide important clinical information to avoid over-treatment or over-diagnosis of diabetes for elderly people.

## 2. Methods

### 2.1. Ethics Statement

The data were collected from the Taiwan Biobank database. The Institutional Review Board on Biomedical Science Research/IRB-BM, Academia Sinica, Taiwan, and the Ethics and Governance Council of Taiwan Biobank, Taiwan, approved the protocol of the Taiwan Biobank. Written informed consent was obtained from each participant. The current study was approved by the Institutional Review Board of Kaohsiung Medical University Hospital (IRB No: KMUHIRB-E(I)-20180242).

### 2.2. Study Participants

The Taiwan Biobank database contains the information of ethnic Taiwanese residents aged 30 to 70 years. After excluding participants with diabetes, a total of 4748 participants (2183 males and 2563 females) between the ages of 30 to 70 years from 2008 to 2014 were included in the final analysis. The venous blood samples were collected and filled in blood collection tubes containing K2EDTA. Not all of these participants were in fast before blood sampling. Among these participants, HbA1c levels were measured from their blood samples.

### 2.3. Statistical Analysis

All statistical analyses were performed using the SPSS software, version 19.0 (IBM SPSS, Armonk, NY, USA). Continuous variables were expressed as mean and standard deviation, and categorical variables were expressed as frequency and percentage. People younger than 50 years have a low incidence of type 2 diabetes [13]. For association analysis, these participants were divided into two age-related groups in which one contained subjects less than 50 years of age and the other contained subjects 50 years or older. The association between HbA1c levels and age was conducted using linear regression analysis. The difference of HbA1c levels between males and females was compared by two-sample *t*-test. To investigate the difference of HbA1c levels among age groups, the included participants were divided into four age groups: 30–39, 40–49, 50–59, and 60–70 years. The difference of HbA1c values among the age groups were compared using one-way ANOVA in which a significant difference was followed with Fisher’s least significant difference (LSD) post hoc test. A *p*-value < 0.05 was considered as statistically significant.

## 3. Results

### 3.1. Characteristics of Participants

After excluding participants with diabetes from the Taiwan Biobank database, a total of 4748 participants were included in this study. These participants included 2183 (46%) males and 2565 (54%) females. The mean age of these participants was 49.1 ± 10.6 years (range: 30–70 years), and the mean level of HbA1c was 5.69 ± 0.61% (range: 4.3–12.7%).

### 3.2. HbA1c Associated with Age

The result of multiple linear regression analysis showed that there was a significant positive correlation between HbA1c levels and age, and levels for male were significantly higher than for females (Table 1); thus, the associations between HbA1c levels and age for males and females should be investigated separately. For males, the result of single linear regression analysis showed that there was a significant positive correlation between HbA1c levels and age for age groups of 30–70 (*p* < 0.0001) and 30–49 (*p* = 0.0003) years, but did not show a significant correlation for the age group of 50–70 (*p* = 0.3567) years (Table 2). For females, there was a significant positive correlation between HbA1c levels and age for all age groups: 30–70 (*p* < 0.0001), 30–49 (*p* < 0.0001), and 50–70 (*p* < 0.0001) years (Table 2).

### 3.3. HbA1c Levels between Sexes among Different Age Groups

To investigate the differences of HbA1c levels between sexes among ages, the participants were divided into four age groups: 30–39, 40–49, 50–59, and 60–70 years. The HbA1c levels of males were significantly higher than females for the two age groups of 30–39 (*p* < 0.0001) and 40–49 (*p* = 0.0017) years (Table 3). However, no significant differences between sexes were observed in the older age groups of 50–59 (*p* = 0.0895) and 60–70 (*p* = 0.7522) years (Table 3). The HbA1c levels among the four age groups showed significant differences for both sexes except for two age groups of 50–59 and 60–70 years in males, and their levels significantly increased with age (Table 3).

### 3.4. HbA1c between Sexes for Different Hba1c Levels

A HbA1c level ≥ 6.5% is suggested as the diagnosis of diabetes. Although the included participants had not been diagnosed with diabetes previously, these may contain participants with HbA1c ≥ 6.5%, so the participants were divided into two groups (HbA1c < 6.5% and HbA1c ≥ 6.5%), and their HbA1c levels for the two groups were investigated separately. For all participants, the HbA1c levels of males were significantly higher than for females (*p* < 0.0001) (Table 4). The same result was also observed for the group of HbA1c < 6.5% (*p* < 0.0001). However, there was no significant difference between sexes for the group with HbA1c ≥ 6.5% (*p* = 0.2504).

### 3.5. HbA1c Associated with Age for Different Hba1c Level Groups

**Figure 1:**
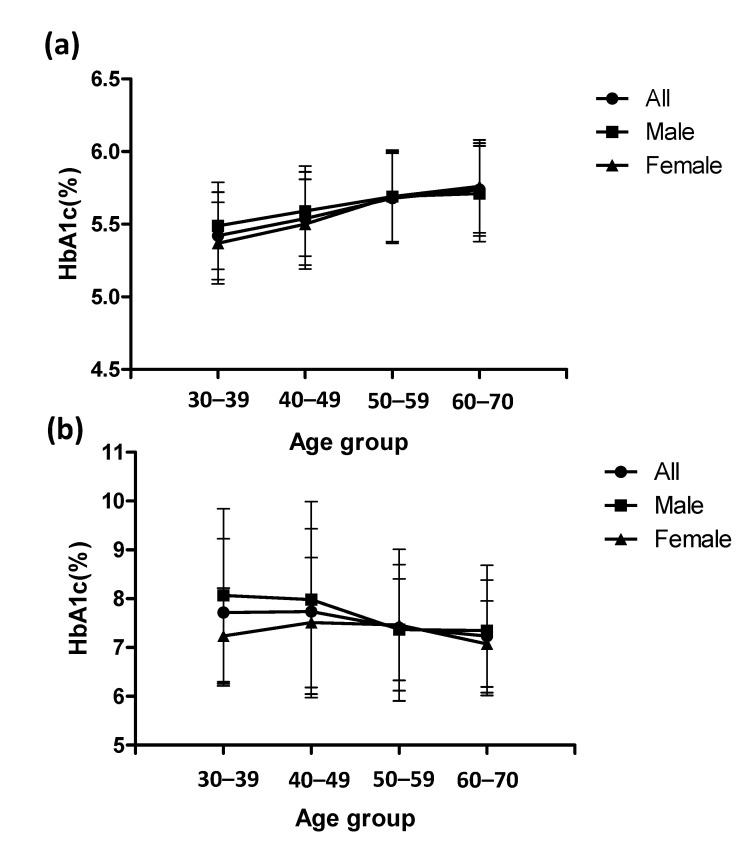
HbA1c levels among different age groups for HbA1c < 6.5% (**a**) and HbA1c ≥ 6.5% (**b**). Error bars indicate ± one standard deviation.

For males with HbA1c < 6.5%, the result of single linear regression analysis showed that there was a significant positive correlation between HbA1c levels and age for all ages (*p* < 0.0001) and the age group of 30–49 (*p* < 0.0001) years, but no significant correlation was observed for the age group of 50–70 (*p* = 0.1583) years (Table 5 and Figure 1). For females with HbA1c < 6.5%, there was a significant positive correlation between HbA1c levels and age for all ages (*p* < 0.0001) and age groups of 30–49 (*p* < 0.0001) and 50–70 (*p* < 0.0001) years (Table 5 and Figure 1). For males with HbA1c ≥6.5%, there was a significant negative correlation between HbA1c levels and age for all ages (*p* < 0.0001) but no significant correlation was observed for age groups of 30–49 (*p* = 0.5248) and 50–70 (*p* = 0.6822) years. For females with HbA1c ≥ 6.5%, no significant correlation was observed for all ages (*p* = 0.2968) and age groups of 30–49 (*p* = 0.4140) and 50–70 (*p* = 0.5616) years (Table 5 and Figure 1).

### 3.6. HbA1c Levels between Sexes for Different Hba1c Level Groups

For participants with HbA1c < 6.5%, the HbA1c levels of males were significantly higher than for females for age groups of 30–39 (*p* < 0.0001) and 40–49 (*p* < 0.001) years (Table 6) but were significantly less than females for the age group of 60–70 years (*p* = 0.0448). For participants with HbA1c ≥6.5%, there were no significant differences between sexes for all age groups (Table 6). For the participants with HbA1c < 6.5%, there were significant differences among age groups for both sexes except for the two age groups of 50–59 and 60–70 years in males (Table 6). For participants with HbA1c ≥ 6.5%, there were no significant differences among age groups for both sexes (Table 6).

## 4. Discussion

HbA1c is an important indicator for the diagnosis of diabetes. The impact of age and gender on HbA1c levels has been investigated for Chinese adults without diabetes [12]. However, these associations have not been investigated for Taiwanese adults without diabetes. The results of the current study showed that HbA1c levels were positively correlated with age, and the levels of males were significantly higher than for females. However, there was no significant positive correlation between HbA1c levels and age in males for the age group of 50–70 years. The HbA1c levels of males were significantly higher than females for the two age groups of 30–39 and 40–49 years. There were significant differences in HbA1c levels among age groups for all participants except for the two age groups of 50–59 and 60–70 years in males. For the participants with HbA1c < 6.5%, the results were similar to those of all participants. For participants with HbA1c ≥ 6.5% however, a significant negative correlation between HbA1c levels and age was observed in males. Moreover, no significant differences in HbA1c levels between sexes were observed in any age groups and no significant differences in HbA1c levels were observed among age groups for participants with HbA1c ≥ 6.5%.

Our results demonstrated that HbA1c levels in males were significantly higher than for females for the two age groups of 30–39 and 40–49 years. A similar result was also observed in a previous study for the age group of 30–59 years in Chinese adults [12]. However, our results showed that there was no significant difference in HbA1c levels between sexes for age group of 50–59 years. This difference between Chinese and Taiwanese adults for the age group of 50–59 years might be due to the differences between Chinese and Taiwanese lifestyles. Menstruating women with rapid erythrocyte turnover have lower hemoglobin levels. This could explain why the HbA1c levels of females were significantly less than for males for the age group of 30–49 years, as suggested previously [7]. Moreover, the mean age at menopause was reported to be 50.2 years for Taiwanese women [14]. This could explain why no significant differences in HbA1c levels between sexes were observed after the age of 50 years for Taiwanese adults.

Our results demonstrated a positive correlation between HbA1c levels and age. Similar results have also been reported for people without diabetes [4,5,6,7,12]. These results indicate that age seems to be an independent factor affecting the HbA1c levels. A previous study has reported that many physiological parameters decrease with age resulting in HbAc1 levels increasing with age, such as tissue sensitivity to insulin, insulin receptor activity, and the function of pancreatic islets [12].

No significantly positive correlation between HbA1c levels for age between 50–70 years in males was observed, but this did not occur in females. Similar results in males could also be observed in Chinese aged ≥70 years [12] and in Japanese aged ≥54 years [15]. However, the reason why the HbA1c levels did not increase with age for older males is unclear.

There are some limitations in this study. Although the Taiwan Biobank database provides relatively large sample size and reliable data, the database lacks participants younger than 30 years and older than 70 years. Further study should collect participants with a wider range in age to investigate the association between HbA1c levels and age. Some factors that might affect HbA1c levels were not excluded from this study and these may produce bias in our results; for example, ranolazine can decrease HbA1c levels [16]; and cardiovascular disease is positively correlated with HbA1c levels for people without diabetes [17]. However, the Taiwan Biobank database does not provide complete medical records and our results might be affected by these confounders. Finally, pregnant women may develop gestational diabetes mellitus [18] and their HbA1c levels could become abnormally high. This would also be another confounding factor affecting our results.

## 5. Conclusions

This study investigated the association between HbA1c levels and age in Taiwanese adults without prior diagnosis with diabetes. In general, the HbA1c levels increased with aging and the levels of males were significantly larger than females. However, the HbA1c levels did not increase with age for the age group of 50–70 years in males. Moreover, the HbA1c levels of males were significantly higher than for females for the age group of 30–49 years. A slight difference in HbA1c levels between Chinese and Taiwanese adults was observed. Our results provide a sex-specific association between HbA1c levels and age, where age and gender were important factors affecting HbA1c levels. Based on our results, the HbA1c cut-off point for the diagnosis of diabetes should vary by age and gender.

## Figures and Tables

**Table 1:** Multiple linear regression of Hemoglobin A1c (HbA1c) values associated with age and gender.

Variable	β	SE	*t*-Value	*p*-Value
Age	0.01401	0.00081	17.25	**<0.0001**
Male (ref: Female)	0.08301	0.01725	4.81	**<0.0001**

β: beta value; SE: standard error; ref: reference. Bold fonts indicate *p*-value < 0.05.

**Table 2:** Single linear regression between HbA1c levels and age.

Age Group	β	SE	*t*-Value	*p*-Value
Male				
All age	0.01082	0.00127	8.54	**<0.0001**
Age: 30–49	0.01246	0.00341	3.66	**0.0003**
Age: 50–70	0.00331	0.00359	0.92	0.3567
Female				
All age	0.01713	0.00103	16.62	**<0.0001**
Age: 30–49	0.01954	0.00236	8.27	**<0.0001**
Age: 50–70	0.01295	0.00312	4.15	**<0.0001**

β: beta value. SE: standard error. Bold fonts indicate *p*-value < 0.05.

**Table 3:** HbA1c levels between sexes for different age groups.

Age Group	All		Male		Female		*p*-Value ^$^
	*N*	Mean ± SD	*n*	Mean ± SD	*n*	Mean ± SD	
30–39	1070	5.47 ± 0.47 ^a^	517	5.55 ± 0.54 ^a^	553	5.40 ± 0.37 ^a^	**<0.0001**
40–49	1316	5.63 ± 0.63 ^b^	583	5.69 ± 0.72 ^b^	733	5.58 ± 0.56 ^b^	**0.0017**
50–59	1429	5.80 ± 0.63 ^c^	632	5.83 ± 0.63 ^c^	797	5.77 ± 0.63 ^c^	0.0895
60–70	933	5.86 ± 0.61 ^d^	451	5.87 ± 0.70 ^c^	482	5.86 ± 0.51 ^d^	0.7522
*p*-value ^&^		**<0.0001**		**<0.0001**		**<0.0001**	

SD: standard deviation. ^$^ The difference between sexes was tested by the two-sample *t*-test. ^&^ The differences among age groups were tested by one-way ANOVA. The same letter indicates no significant difference. Bold fonts indicate *p*-value < 0.05. HbA1c levels are expressed as percentages.

**Table 4:** HbA1c levels between sexes for HbA1c < 6.5% and HbA1c ≥ 6.5%.

Group	All		HbA1c < 6.5%		HbA1c ≥ 6.5%	
	*n*	Mean ± SD	*n*	Mean ± SD	*n*	Mean ± SD
Male	2183	5.73 ± 0.66	2050	5.62 ± 0.32	133	7.54 ± 1.44
Female	2565	5.65 ± 0.57	2449	5.57 ± 0.34	116	7.34 ± 1.29
*p*-value		**<0.0001**		**<0.0001**		0.2504

SD: standard deviation. Bold fonts indicate *p*-value < 0.05.

**Table 5:** Single linear regression between HbA1c levels and age.

Variable	β	SE	*t*-Value	*p*-Value
HbA1c < 6.5% for male				
All age	0.00757	0.00063	12.06	**<0.0001**
Age: 30–49	0.00782	0.00167	4.68	**<0.0001**
Age: 50–70	0.00253	0.00179	1.41	0.1583
HbA1c < 6.5% for female				
All age	0.01413	0.00060	23.46	**<0.0001**
Age: 30–49	0.01386	0.00148	9.38	**<0.0001**
Age: 50–70	0.01017	0.00175	5.82	**<0.0001**
HbA1c ≥ 6.5% for male				
All age	−0.03160	0.01336	−2.36	**0.0195**
Age: 30–49	−0.03960	0.06165	−0.64	0.5248
Age: 50–70	−0.00945	0.02301	−0.41	0.6822
HbA1c ≥ 6.5% for female				
All age	−0.01446	0.01379	−1.05	0.2968
Age: 30–49	−0.03813	0.04610	−0.83	0.414
Age: 50–70	−0.01529	0.02623	−0.58	0.5616

β: beta value; SE: standard error. Bold fonts indicate *p*-value < 0.05.

**Table 6:** HbA1c levels of different age groups between sexes for HbA1c < 6.5% and HbA1c ≥ 6.5%.

Age Group	All		Male		Female		*p*-Value ^$^
	*n*	Mean ± SD	*n*	Mean ± SD	*n*	Mean ± SD	
HbA1c < 6.5%							
30–39	1051	5.42 ± 0.30 ^a^	506	5.49 ± 0.30 ^a^	545	5.37 ± 0.28 ^a^	**<0.0001**
40–49	1262	5.54 ± 0.32 ^b^	557	5.59 ± 0.31 ^b^	705	5.50 ± 0.31 ^b^	**<0.0001**
50–59	1330	5.68 ± 0.31 ^c^	578	5.69 ± 0.31 ^c^	752	5.69 ± 0.32 ^c^	0.4125
60–70	856	5.74 ± 0.32 ^d^	409	5.71 ± 0.33 ^c^	447	5.76 ± 0.32 ^d^	**0.0448**
*p*-value ^&^		**<0.0001**		**<0.0001**		**<0.0001**	
**HbA1c ≥ 6.5%**							
30–39	19	7.72 ± 1.51	11	8.07 ± 1.77	8	7.24 ± 0.98	0.2456
40–49	54	7.74 ± 1.69	26	7.98 ± 2.01	28	7.51 ± 1.33	0.3167
50–59	99	7.41 ± 1.29	54	7.37 ± 1.04	45	7.46 ± 1.55	0.7407
60–70	77	7.23 ± 1.15	42	7.35 ± 1.33	35	7.07 ± 0.88	0.2754
*p*-value ^&^		0.153		0.145		0.487	

SD: Standard deviation. ^$^ The difference between sexes was tested by the two-sample *t*-test. ^&^ The differences among age groups were tested by one-way ANOVA. The same letter indicates no significant difference among age groups. Bold fonts indicate *p*-value < 0.05.

## Data Availability

The data underlying this study is from the Taiwan Biobank. Due to restrictions placed on the data by the Personal Information Protection Act of Taiwan, the minimal data set cannot be made publicly available.
